# A rapid response-type doctor car system shortened time to intravenous thrombolytic therapy for patients with ischemic stroke: an observational study at a single emergency center in Japan

**DOI:** 10.1186/s12245-020-00292-y

**Published:** 2020-06-26

**Authors:** Yuki Yoshioka, Mina Gamo, Ryuhei Yoneda, Naoki Matsunaga, Tadaaki Takada, Yasushi Fukuta, Koichi Sato

**Affiliations:** 1 Department of Emergency & Critical Care Medicine, Tokushima Red Cross Hospital, 103, Irinoguchi, Komatsushima-cho, Komatsushima City, Tokushima 773-8502 Japan; 2 Department of Neurosurgery, Tokushima Red Cross Hospital, 103, Irinoguchi, Komatsushima-cho, Komatsushima City, Tokushima 773-8502 Japan

## Abstract

**Background:**

For patients with ischemic stroke, rapid reperfusion therapy is extremely important. In April 2015, our medical center introduced a rapid response-type doctor car (RRC) system. Here, an emergency medical team, including a physician, is dispatched to the patient’s prehospital location. The team then assesses the patient and, if necessary, initiates infusion therapy, excluding thrombolytic therapy. Before arriving at the hospital, a prehospital physician orders the preparation of diagnostic tools and conducts an early consultation to a neurologist in order to begin thrombolytic therapy more swiftly. This study aimed to determine whether the RRC system shortened the time to commence intravenous reperfusion therapy in patients with ischemic stroke. This was a retrospective observational study conducted at a tertiary emergency center in Japan. Cases of patients with ischemic stroke who underwent intravenous thrombolytic therapy from January 2015 to December 2018 were enrolled. They were divided into two groups: RRC group (intervened by RRC system) and non-RRC group (not intervened by RRC system). The groups’ door-to-needle (DTN) time was compared.

**Results:**

During the study period, 140 patients received intravenous thrombolytic therapy. Among those, 28 were in the RRC group and 28 received the usual prehospital care. Of 56 patients, the median age was 82 years old, and 42.9% of patients were male. The median NIHSS was 14 (IQR 10–21). As for demographics, there were no significant differences between the two groups. Median DTN time was 67 min (IQR 55–79) in RRC group vs. 81 min (IQR 69–107) in usual care group, respectively (*P* < 0.05).

**Conclusion:**

In this study, patients with ischemic stroke in RRC group received intravenous thrombolytic therapy in a shorter time compared to the group that received usual care.

## Background

In patients with ischemic stroke, rapid reperfusion therapy is extremely important [1]. Various interventions have been tried in order to shorten the time to commence thrombolytic therapy for ischemic stroke, such as a physician-staffed helicopter service [2] and ambulance-based thrombolysis [3, 4]. However, the results varied, and the applicability to the emergency medical system was unclear. Furthermore, for most emergency medical services, expensive proposals such as adding computed tomography (CT) capabilities to an ambulance are not practical.

In April 2015, our center introduced a rapid response-type doctor car (RRC) system in which the emergency medical team includes an emergency physician and nurse. This team is dispatched to the patient’s site, where its members assess the patient and begin to prepare for diagnostics and treatment after the arrival at the hospital. Unlike proposals requiring special equipment such as a CT or laboratory tests, our system involves only manpower, making it potentially less expensive and, perhaps, more easily adaptable in other countries.

In this study, we examined the effect of an RRC system on the time to commence thrombolytic therapy for the patients with ischemic stroke.

## Methods

This was a retrospective observational study conducted at a tertiary emergency center in Japan. Our RRC system is activated when an emergency call suggests severe injury or illness, including acute stroke. The medical team on an RRC is dispatched from our hospital simultaneously with an ambulance staffed by an emergency medical service (EMS) crew from a fire department nearby a patient. The RRC vehicle utilizes a sport utility vehicle that lacks patient transport capabilities, so the patient is transported by an ambulance. The RRC system runs from 9:00 am to 7:00 pm on weekdays and from 9:00 am to 5:00 pm on weekends. Generally, the distance the RRC runs from our hospital is approximately at a 15-km radius to patient homes. The population of the area where the RRC covers is approximately 200 thousand people.

In patients who underwent intravenous thrombolytic therapy for ischemic stroke, some patients were transported by only emergency medical services (EMS), while others received an intervention through the RRC system, and some were transferred to our center from other hospitals. In Japan, EMS crews cannot begin intravenous saline infusions in a patient without assessing for a shock status and are not permitted to administer any cardiovascular drugs, including antihypertensive agents. They can only check the blood glucose levels of comatose patients. For patients with suspected stroke, the prehospital priority of EMS crew is for rapid transport and not for intervention.

As for patients transferred to our center from other hospitals, most patients receive saline infusion. Before arriving at our hospital, the doctor of other hospitals will begin a consult with a neurologist in our institute. Thus, emergency physicians are not involved in treating patients from other institutions, and only neurologists engage in the inspection and treatment.

On the other hand, in the RRC system, the field physician and the nurse rule out hypoglycemia, and start a prehospital saline infusion. Assessment and infusion are rapidly performed, so that the median time that an ambulance stops at the site is only 4 min. This length of time adds to the duration prior to arrival at the hospital, but with the physician’s effort to initiate assessment, management time is shortened by the RRC system. During transport, the field physician contacts another emergency physician in the emergency department to report the patient’s status and facilitate consultation with neurology. The intent is to speed up the process of assessment and to curtail time to therapy once the patient arrives at the hospital.

After arrival at our center, the strategy of diagnosis is the same regardless of the prehospital interventions. The patients with suspected cerebral infarction first undergo brain magnetic resonance imaging, unless they are comatose or repeatedly vomiting, in which case, brain CT is the initial study of choice. After imaging and assessment, a neurologist decides whether to begin intravenous thrombolytic therapy. This therapy generally starts in an intensive care unit.

In our retrospective study, we enrolled patients with ischemic stroke, who underwent intravenous thrombolytic therapy from January 2015 to December 2018. They were divided into two groups: those who received RRC care (RRC group) and those who received the usual prehospital care (non-RRC group). Because the RRC system is not available round-the-clock, the non-RRC group consisted only of patients who underwent thrombolytic therapy during RRC operational hours. The non-RRC group did not include patients who walked to the emergency department. As described above, the non-RRC group included patients transported by only the EMS crew from the patient’s site and the patients transferred from other hospitals.

Demographic and clinical data included the age, sex, National Institute of Health Stroke Scale (NIHSS) [5], and results of initial imaging studies. The primary outcome was the time to commence intravenous thrombolytic therapy and the time from hospital arrival to commencement of intravenous thrombolytic therapy (door-to-needle [DTN] time). We compared DTN time between the two groups. Secondary outcomes were the time from symptom onset to commencement of intravenous thrombolytic therapy (onset-to-needle [OTN] time), mortality rate, modified Rankin scale (mRS) [6], and Barthel Index (BI) for activities of daily living upon hospital discharge. We reported the proportion of patients with mRS of 0 to 3, which were considered good neurological outcomes.

This study was approved by our center’s Ethics Committee. Statistical analysis was performed using R (R studio version 1.1.456, running R 3.5.1). Continuous variables are described as medians and interquartile ranges (IQR). Comparisons between two groups with nonparametric data were made by the Wilcoxon signed-rank test. Comparisons for categorical variables were performed using Fisher’s exact test. We considered the *P* values of < 0.05 to be statistically significant.

## Results

**Fig. 1:**
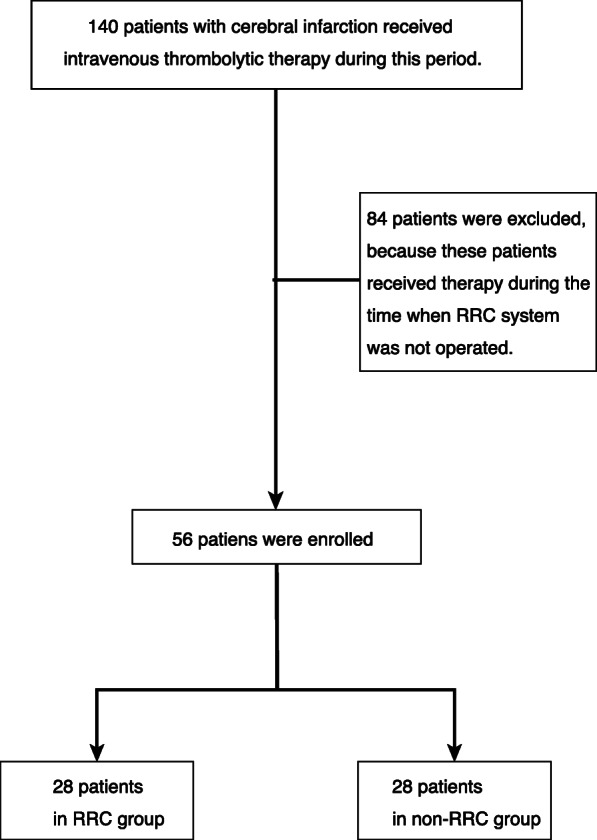
Flowchart of patients

During the study period, 140 patients received intravenous thrombolytic therapy. Of these, 28 received the RRC intervention, and 28 received the usual care while the RRC system was operating (Fig. 1). The demographics of the enrolled patients are described in Table 1. The median age was 82 years old, and 42.9% of patients were male. The median NIHSS was 14 (IQR 10–21). As for demographics, there were no significant differences between the two groups. With regard to the primary outcome, the median DTN in the RRC and non-RRC group was 67 min (IQR 55–79) vs. 81 min (IQR 66–101), respectively (*P* < 0.05) (Table 2). The secondary outcomes of mortality, mRS, and BI did not significantly differ between groups. As to OTN, the median OTN in RRC group and non-RRC group was 139 min (IQR 113–165) vs. 168 min (IQR 127–215), respectively (*P* < 0.05).

**Table 1:** Demographics of the patients

	Overall	RRC	Non-RRC	*P* value
Number	56	28	28	
Age	82 (71–90)	81 (71–86)	82 (72–91)	0.46
Sex	42.9% male	42.9% male	42.9% male	1
NIHSS	14 (10–21)	14 (12–20)	14 (9–22)	0.55
MRI*	91.1%	89.3%	92.3%	0.39
EVT**	19.6%	17.9%	21.4%	1

^*^The rate of the patients who were firstly inspected by cerebral MRI

^**^EVT=endovascular thrombectomy

**Table 2:** Outcomes of this study

	Overall	RRC	Non-RRC	*P* value
DTN (min)	72 (60–94)	67 (55–79)	76 (66–101)	< 0.05
OTN (min)	144 (117–185)	139 (113–165)	168 (127–215)	< 0.05
mRS (0–3)*	32.1%	28.6%	35.7%	0.49
BI	15 (0–61)	40 (0–61)	0 (0–95)	0.36
Mortality (%)	5.4%	0%	10%	0.24

^*^The rate of the patients with mRS from zero to three

## Discussion

Immediate reperfusion therapy is central to the treatment of ischemic stroke. In this study, we examined the efficacy of our center’s RRC intervention to shorten the time to commence intravenous thrombolytic therapy. Compared with the non-RRC group, the RRC group experienced a significantly shorter time from hospital arrival to thrombolytic therapy.

Numerous medical professionals support our RRC system. Emergency call center personnel dispatch the RRC team, basing this decision on keywords, such as “suddenly collapsed,” “sudden difficulty in speaking,” and “sudden upper and lower limb weakness.” An ambulance crew is simultaneously dispatched with the RRC. This crew collaborates on-site with the RRC physician and nurse in an ambulance. They collect crucial elements of the patient’s history, such as time of onset of symptoms, past medical history, and prescribed medications. The RRC team’s primary task is to assess patients in the prehospital setting, determining the likelihood of ischemic stroke and the patient’s eligibility for intravenous thrombolytic therapy. This information is transmitted to the in-hospital emergency physician and neurologist. Cooperation among the EMS crew, the field physician, and the nurse allowed for the possibility of accelerating the commencement of thrombolytic therapy in the RRC group.

In our district, EMS crews cannot directly contact neurologists. However, it is doubtful whether the action of the EMS crew to contact a neurologist directly can shorten the commencement to thrombolytic therapy compared with our RRC system in our district. The reason is because in this study the patients that were transferred from other hospitals were directly referred to a neurologist by doctors in other hospitals. After arrival of the patient, neurologist promptly assessed the patients suspected with ischemic stroke and began the thrombolytic therapy. This method is similar to direct contact to a neurologist from the EMS crew. However, even direct consultation from the doctor to neurologists in transferred patients, which were included in non-RRC group, did not contribute to a shorter DTN time compared with RRC group. The collaboration between emergency physicians and neurologists after the arrival of the patients is considered to be one of the most important components of our RRC system. In order to take this into account, we enrolled the patients transferred from other hospitals in this study.

In Japan, EMS staff is permitted to provide restricted medical practice compared to that of other countries [7]. Thus, the RRC system, with the primary aim of bringing medical staff, could be effective in Japan. However, the essential contribution of the RRC system is not a particular therapeutic intervention. Rather, it is the assessment of the patients with suspected ischemic stroke to accelerate examination time and, ultimately, reduce the time to commence treatment upon hospital arrival.

In this study, OTN was compared between groups as a secondary outcome. There was a statistically significant difference of OTN between the groups, specifically 139 min (IQR 113–165) in the RRC group vs. 168 min (IQR 127–215) in the non-RRC group. However, the RRC system could not improve the time from onset of symptom to the emergency call for an ambulance. This OTN difference might be a coincidence. The RRC system offered the possibility of shortening the DTN time. Of course, this shortened DTN time in the RRC group somehow contributed to the difference of OTN between the two groups.

To the best of our knowledge, few studies have assessed the efficacy of physician-staffed ambulances for patients with suspected ischemic stroke. Several studies of physician-staffed helicopter EMS (HEMS) proved that these interventions were effective for these patients [2, 8, 9]. However, the essential contribution of HEMS is generally for long-distance transportation cases. HEMS’ effect on DTN was not well-examined. Ground EMS (GEMS) is commonly used for cases of short distance transportation. Our RRC system of physician-staffed GEMS is used for short distances and can be useful for relatively populated areas. The efficacy of physician-staffed GEMS on treating ischemic stroke patients merits further examination.

This study had several limitations. First, it was a retrospective observational study conducted in a single tertiary emergency center in Japan. Our RRC system is unusual even for Japan, probably due to a lack of evidence supporting physician-staffed ground emergency medical systems and a shortage of emergency physicians. Second, the patients enrolled into this study had heterogeneity. In the non-RRC group, some patients were transported by only EMS crews. In such cases, in spite the patient being attended to during RRC operational time, RRC system was not activated. This may be a problem in terms of the skill of the call center personnel in deciding on the activation of the RRC system. Or, this inactivation might result from atypical symptoms of patients. In the present study, the symptoms and chief complaints were not assessed; hence, this probable heterogeneity of the patients might be a confounder of this study. The third limitation is generalizability. EMS and medical systems vary widely among countries. Thus, our study’s results may not apply to other countries. However, the study did find that prehospital assessment by a dedicated physician and nurse can speed treatment time for patients with suspected ischemic stroke. In each country, emergency physicians and neurologists should be able to consider how the medical resources of the country and local districts will focus on the stroke patients.

This study had a small sample size and is only an observational study. We could not show the effect of RRC system on mortality and neurological outcome, such as mRS and BI. We would like to keep on collecting cases, and in the future, to try to examine the effect of RRC system on such outcomes. Furthermore, our RRC system is not operational throughout all the hours of the day. Thus, emergency physicians and neurologists will have to guide EMS crews to precisely assess the patients suspected with stroke, collect the necessary information, and contact in-hospital physicians swiftly. We believe that our RRC system contributes to the improvement of quality of EMS crews’ care during the times when the RRC system would not be operational. This aspect of our RRC system’s contribution is considered to be an important outcome.

## Conclusion

In the present study, patients with ischemic stroke in the RRC group received intravenous thrombolytic therapy in a significantly shorter time compared with usual care group. The focus on manpower, including the EMS crew and medical staff, and the cooperation of emergency physicians and neurologists were considered to be the key to shortening the commencement of thrombolytic therapy.

## Data Availability

All data generated or analyzed during this study are included in this published article.

## Abbreviations

RRC: Rapid response car
OTN: Onset-to-needle time
DTN: Door-to-needle time
IQR: Interquartile range
CT: Computed tomography
EMS: Emergency medicine service
MRI: Magnetic resonance imaging
NIHSS: National Institute of Health Stroke Scale
mRS: Modified Rankin scale
